# UV-LED projection photolithography for high-resolution functional photonic components

**DOI:** 10.1038/s41378-021-00286-7

**Published:** 2021-08-17

**Authors:** Lei Zheng, Urs Zywietz, Tobias Birr, Martin Duderstadt, Ludger Overmeyer, Bernhard Roth, Carsten Reinhardt

**Affiliations:** 1 Hannover Centre for Optical Technologies, Leibniz University Hannover, Hannover, Lower Saxony 30167 Germany; 2 Cluster of Excellence PhoenixD (Photonics, Optics, and Engineering-Innovation Across Disciplines), Hannover, Lower Saxony 30167 Germany; 3 Laser Zentrum Hannover e.V, Hannover, Lower Saxony 30167 Germany; 4 Institute of Transport and Automation Technology, Leibniz University Hannover, Garbsen, Lower Saxony 30823 Germany; 5 Bremen University of Applied Science, Bremen, Bremen, 28199 Germany

**Keywords:** Micro-optics, Nanoscale devices, Other photonics

## Abstract

The advancement of micro- and nanostructuring techniques in optics is driven by the demand for continuous miniaturization and the high geometrical accuracy of photonic devices and integrated systems. Here, UV-LED projection photolithography is demonstrated as a simple and low-cost approach for rapid generation of two-dimensional optical micro- and nanostructures with high resolution and accuracy using standard optics only. The developed system enables the projection of structure patterns onto a substrate with 1000-fold demagnification. Photonic devices, e.g., waveguides and microring resonators, on rigid or flexible substrates with varied geometrical complexity and overall structure dimensions from the nanometer to centimeter scale were successfully prepared. In particular, high-resolution gratings with feature sizes down to 150 nm and periods as small as 400 nm were realized for the first time by this approach. Waveguides made of doped laser active materials were fabricated, and their spontaneous emission was detected. The demonstrated superior performance of the developed approach may find wide applications in photonics, plasmonics, and optical materials science, among others.

## Introduction

Driven by the increasing demand for miniaturization and compact integration of production, advanced fabrication techniques based on lithography have been developed extensively in the past few decades to realize optical and photonic devices with downsized dimensions and high accuracy. To date, highly accurate micro- and nanoelements, e.g., waveguides^1–3^, gratings^4–6^, ring resonators^7–9^, splitters^10,11^, and optical switches^12–14^ that constitute advanced integrated circuits or networks, can be prepared by two-photon lithography^15–17^, electron beam lithography^18–20^, ion beam lithography^21,22^, or nanoimprint lithography^23,24^ on a laboratory scale. Fabrication systems based on the first three methods exhibit excellent performance in high-quality and high-resolution structuring, but they are significantly expensive and limited by low throughput. Nanoimprint lithography enables high-resolution structuring with low cost and high throughput. However, the precise molds required for replication limit its flexibility and necessitate additional financial and time investments^25^. Another technique, extreme ultraviolet (EUV) lithography, produces very high-quality and high-resolution structures. The state-of-the-art smallest feature size is below 10 nm^26,27^. However, this technique is mainly used for mass production of microelectronic integrated chips, and the commercial EUV lithography system costs more than a hundred million euros, which is extremely expensive. Laboratory-scale optical contact lithography^28,29^ and projection photolithography^30–33^ are also being developed for micro- and nanostructuring, but these methods have only achieved resolutions at the micrometer scale thus far. In addition, optical contact lithography requires the use of a contact mask aligner that is expensive and the resolution and precision of the pattern on a photomask are the same as those produced on the target structure, since the pattern is directly transferred onto the substrate without demagnification. The currently available projection lithography usually employs a mercury lamp as the light source, in which case a bulky cooling system is required and a higher cost results.

To improve the production capabilities of optical projection lithography at the micro- and nanoscale and reduce the cost and size of fabrication instruments, we present UV-LED microscope projection photolithography (MPP), a fabrication approach using light to transfer a pattern to a substrate by use of a standard microscope objective; this is a powerful alternative technology for the fabrication of high-quality photonic components. A simple and low-cost UV-LED MPP setup was developed as an advancement, and its capabilities in the rapid and precise generation of high-resolution 2D micro- and nanostructures are demonstrated. In the developed MPP setup, tube lenses were implemented and combined with the objective to create an infinity-corrected optical system for the purpose of minimizing imaging aberrations, which significantly affect the quality of fabricated structures. Autofocus and stitching processes were developed to enable automation of the system and realize large-area structuring. With this fabrication approach, high-resolution diffractive gratings with feature sizes down to 150 nm and periods of 400 nm were realized for the first time. Moreover, large-area microstructures at the millimeter and centimeter scales, elements on both rigid and flexible substrates, and microstructures made of photopolymers with embedded laser active material, which have not been reported, were successfully fabricated. In particular, the use of polymers doped with laser active material opens the route to the creation of novel active photonic devices for various applications. The obtained results clearly show that the developed MPP approach is a powerful tool for rapid, flexible, and high-resolution optical fabrication.

## Results

Using the developed MPP approach, optical micro- and nanostructures were realized by following three main steps, which cover the procedures from structure design to fabrication of the structures on substrates. Two setups comprising Tessar projection photolithography (TPP) and MPP were developed for the preparation of patterned chromium photomasks and the fabrication of the desired structures, respectively. A detailed description and schematic illustrations of both systems are given in the Materials and methods section. To demonstrate the capabilities of the approach, various micro- and nanostructures with different geometries, dimensions, substrates, and materials were produced.

### Single-mode straight and crossed waveguides

**Fig. 1:**
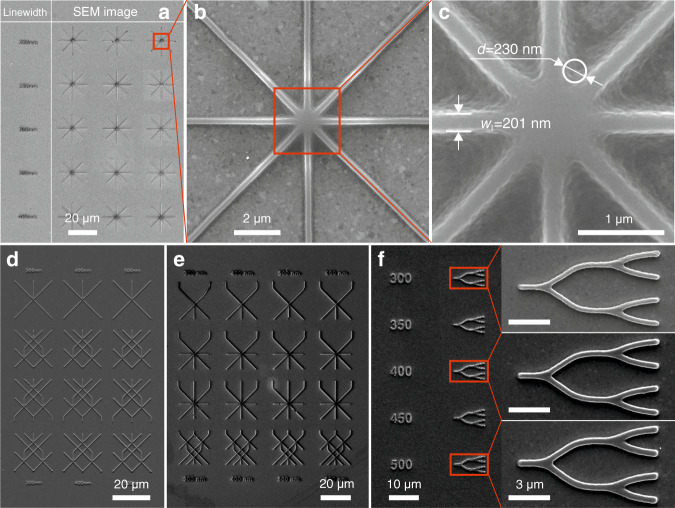
SEM images of single-mode crossed waveguides fabricated on a gold film. **a** Crossed waveguides with different widths *w*_l_. **b** Crossed waveguides with a designed width of 200 nm. **c** Magnified view of waveguides (**b**) and crossing area defining the cross resolution. **d, e** Waveguides with different feature sizes and levels of complexity. **f** Structures with curved features and different feature sizes.

Single-mode waveguides with varied geometrical complexity were fabricated in a first step (Fig. 1), which contains a group of single-mode crossed waveguides with different geometries and feature sizes. The structures are made of a self-synthesized low-shrinkage organic–inorganic hybrid photosensitive material^34^ and were fabricated on a 50 nm thick gold film, which was deposited on a glass substrate. A 100x objective with a numerical aperture (NA) of 1.4 was used for the structuring.

From the top to the bottom of the structure array shown in Fig. 1a, the designed width (*w*_l_) of the waveguides is increased from 200 nm to 400 nm in steps of 50 nm. Three structures were reproduced for each size, indicating high reproducibility. The structure formation accuracy is obvious from the SEM image of single waveguides with a designed width of 200 nm (Fig. 1b). The fabricated waveguides exhibit a smooth surface topography, as seen in the magnified SEM image of the crossed waveguides in Fig. 1c. Additionally, the linewidth and the cross resolution of the waveguides at the intersection area were measured. A linewidth of approximately 201 nm was obtained by measuring the full width at half maximum of the profile. In comparison to the design width, this demonstrates the capability of MPP for highly precise fabrication. A cross resolution, which is defined as the diameter (*d*) of a circle that accurately approximates the area between the intersecting structures, of approximately *d* = 230 nm was obtained.

Furthermore, crossed waveguides with feature sizes at the nanoscale were produced. Both Fig. 1d and Fig. 1e show groups of waveguides with increasing geometrical complexity. For a given geometry, structures with different feature sizes were achieved. Additionally, a so-called complexer that can be used as a light splitter was fabricated with feature sizes in the range between 300 and 500 nm (Fig. 1f). The panels on the right side are SEM images of single complexer structures. Again, high-quality structure formation and high reproducibility can be confirmed.

### High-resolution diffractive gratings

**Fig. 2:**
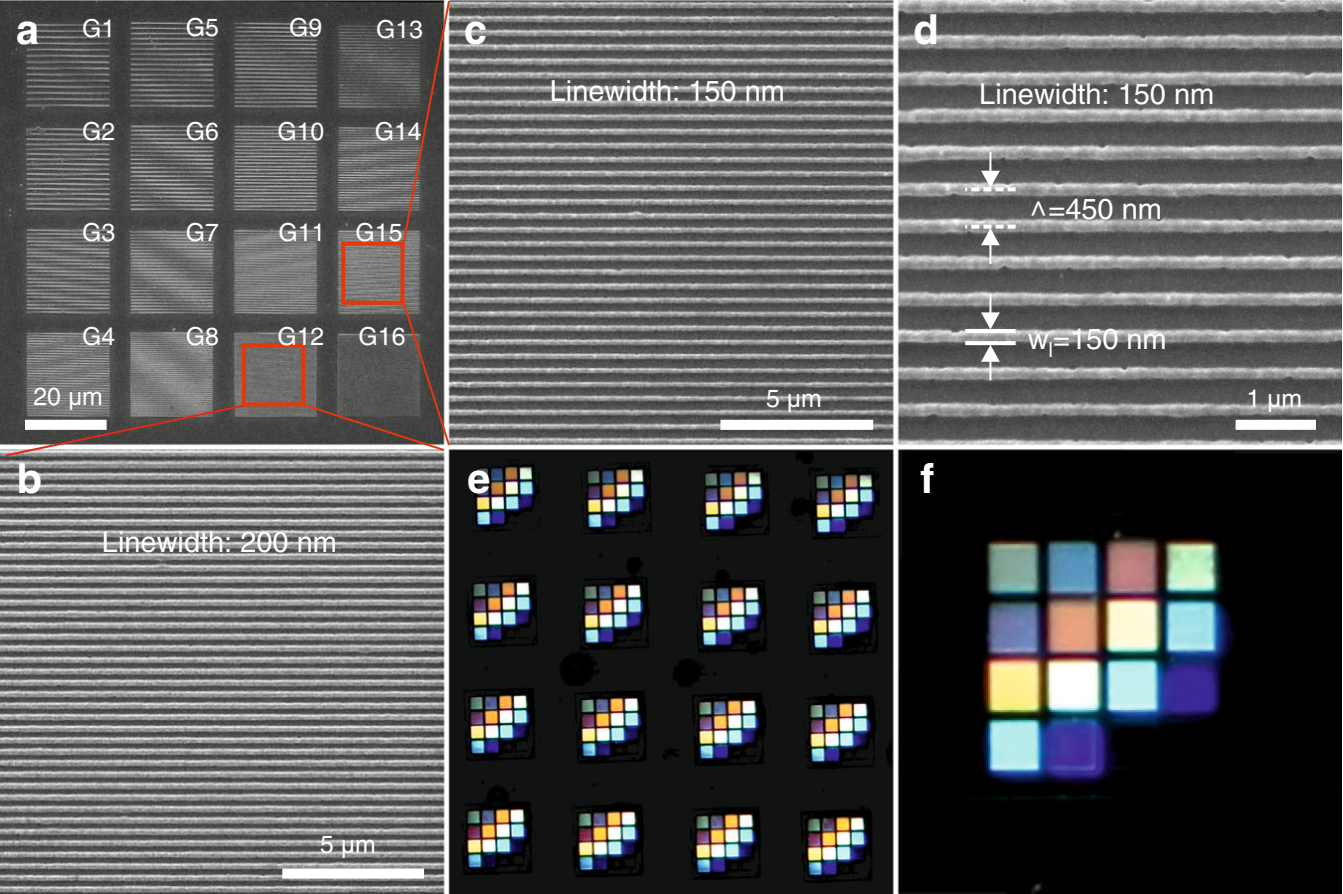
Grating arrays fabricated on glass. **a** SEM image of a grating array. **b** SEM image of the grating with a linewidth of 200 nm and a period Λ = 400 nm. **c** SEM image of the grating with a linewidth as small as 150 nm and a period Λ = 450 nm. **d** Magnified view of the grating in (**c**). **e** Microscope image of the diffraction pattern observed when the grating arrays are illuminated by a white light source in the direction perpendicular to the substrate surface. **f** Microscope image of the diffraction pattern of a single grating array.

High-resolution diffractive grating arrays with a single grating size of 20 μm × 20 μm and different periods Λ and linewidths *w*_l_ were fabricated on glass substrates (Table 1 and SEM image in Fig. 2a). The period Λ denotes the center-to-center distance between two adjacent features. The grating labeling given in Table 1 corresponds to the one marked on the manufactured structures shown in Fig. 2a. The photopolymer used for fabrication is a self-synthesized organic–inorganic hybrid photosensitive polymer^34^. A 100x oil immersion microscope objective with a NA of 1.4 was applied for the fabrication, and the exposure time for the structuring process was 1.5 s. Well-positioned arrays without tilt along the direction perpendicular to the substrate were observed. Additionally, magnified observations of the fabricated gratings were performed. For instance, Fig. 2b–d shows magnified SEM images of fabricated grating samples G12 and G15, respectively. Grating G12 has a period of 400 nm and a linewidth of 200 nm, and G15 exhibits a period of 450 nm and a linewidth of 150 nm.

**Table 1:** Dimensions designed for each grating in the array.

Grating	*w*_*l*_ [nm]	Λ [nm]	Grating	*w*_*l*_ [nm]	Λ [nm]	Grating	*w*_*l*_ [nm]	Λ [nm]	Grating	*w*_*l*_ [nm]	Λ [nm]
G1	300	1500	G5	250	1250	G9	200	1000	G13	150	750
G2	300	1200	G6	250	1000	G10	200	800	G14	150	600
G3	300	900	G7	250	750	G11	200	600	G15	150	450
G4	300	600	G8	250	500	G12	200	400	G16	150	300

By illuminating the grating arrays using a white light source with an incident direction perpendicular to the substrate surface, a diffraction pattern was observed (Fig. 2e). All grating arrays in Fig. 2e are replications of the array in Fig. 2a. The microscope image (Fig. 2f) presents the diffraction of a single array. The gratings with different periods reveal different colors, while those with identical periods exhibit the same color. For example, gratings G4, G11, and G14 with a period Λ = 600 nm display turquoise coloring. This shows that the fabricated gratings diffract and split white light into different beams that travel in distinct directions, depending on the period of the gratings and the wavelength of the light.

### Large-area single-mode structures with area sizes at the millimeter scale

**Fig. 3:**
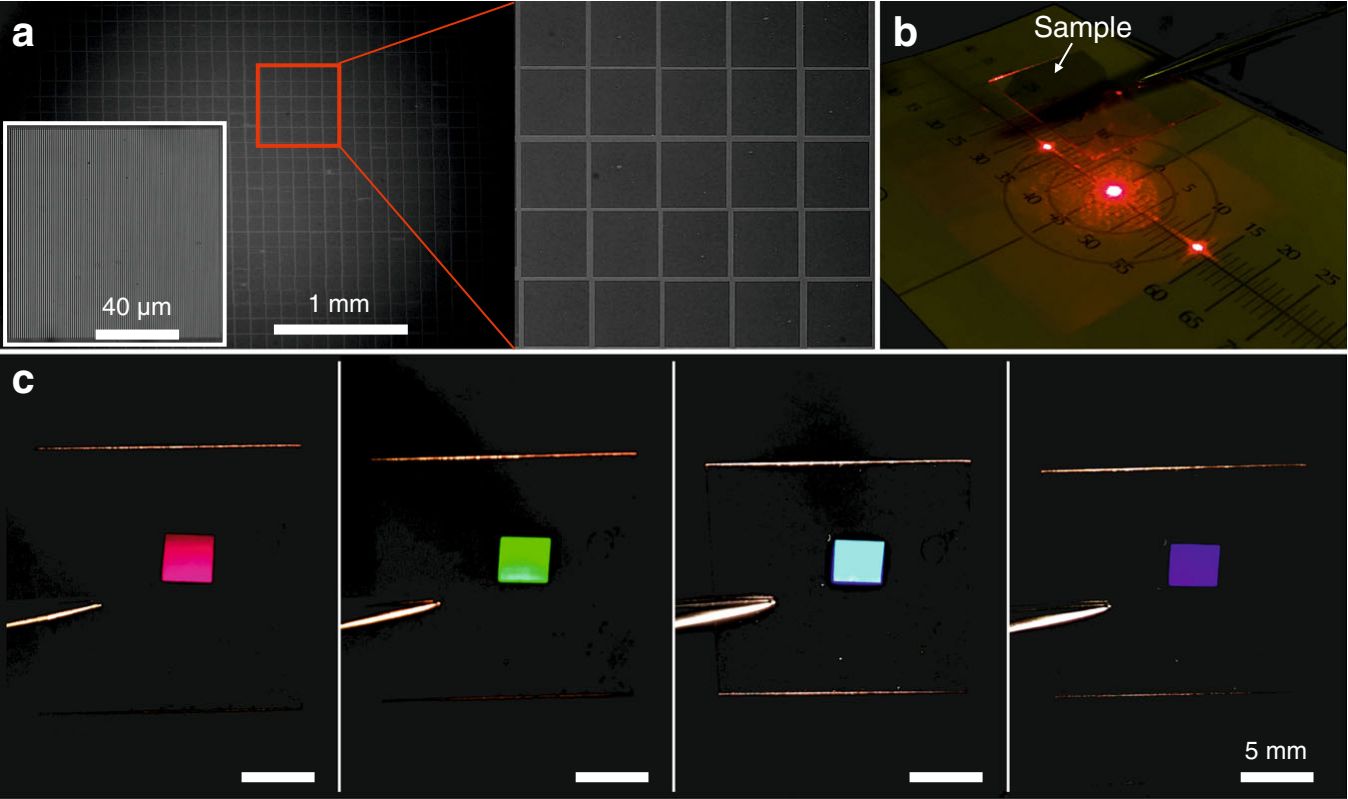
Large-area structures fabricated on a glass substrate. **a** Microscope image of a large-area structure realized through a stitching process. The area size is 3.3 mm × 3.3 mm. **b** Diffraction pattern observed when the sample was illuminated by a He–Ne laser at 633 nm. **c** Images showing the variation of structural coloration of the large-area structure.

Apart from high-resolution gratings, large-area gratings (Fig. 3a, microscope image) with an area of 3.3 mm × 3.3 mm were fabricated via the stitching of a reference grating with dimensions of 100 μm × 100 μm, linewidth *w*_l_ =300 nm, and period Λ = 900 nm. The panel on the lower left in Fig. 3a is a microscope image of the single grating used in the stitching process. The image on the right is an enlarged view of the stitched gratings. Here, an area with a 5 × 5 grating pattern is shown, indicating that the stitching process worked properly. The observed displacement of gratings in the array is due to the lower precision of the motorized stages employed. This can be improved by use of stages with more precise step movements. Simple diffraction experiments were also carried out by illuminating the samples with a He–Ne laser at 633 nm and observing the diffraction (Fig. 3b). The first diffraction modes are clearly seen in the figure. This also results from the grating equation Λ(sin *θ*_m_ – sin *θ*_i_) = *mλ*, where Λ indicates the center-to-center distance of adjacent lines, *θ*_m_ is the maximum diffraction angle between the diffracted wave and the normal vector of the grating, *θ*_i_ is the incident angle of the laser beam, *m* is a nonzero integer representing the diffraction mode of interest, and *λ* is the wavelength of the incident light. In this experiment, Λ = 900 nm, *θ*_i_ = 0, and *λ* = 633 nm; thus, $\sin \theta _m = \frac{{m\lambda }}{\Lambda } \approx 0.7m$ is obtained. Considering –1 ≤ sin *θ*_m_ ≤ 1, *m* = ±1, and *θ*_i_ = 44.7° can be deduced. Additionally, diffraction of light by the fabricated structures was also observed by rotating the sample under a white light source, which illuminates the sample along a direction perpendicular to the horizontal plane. Consequently, variations in the structural coloration resulting in magenta, green, turquoise, and blue (Fig. 3c) were observed with the sample continuously rotating around the horizontal axis. This variation occurred due to changes in the incident angle following rotation of the sample.

### Multimode waveguides at the micrometer and centimeter scales

**Fig. 4:**
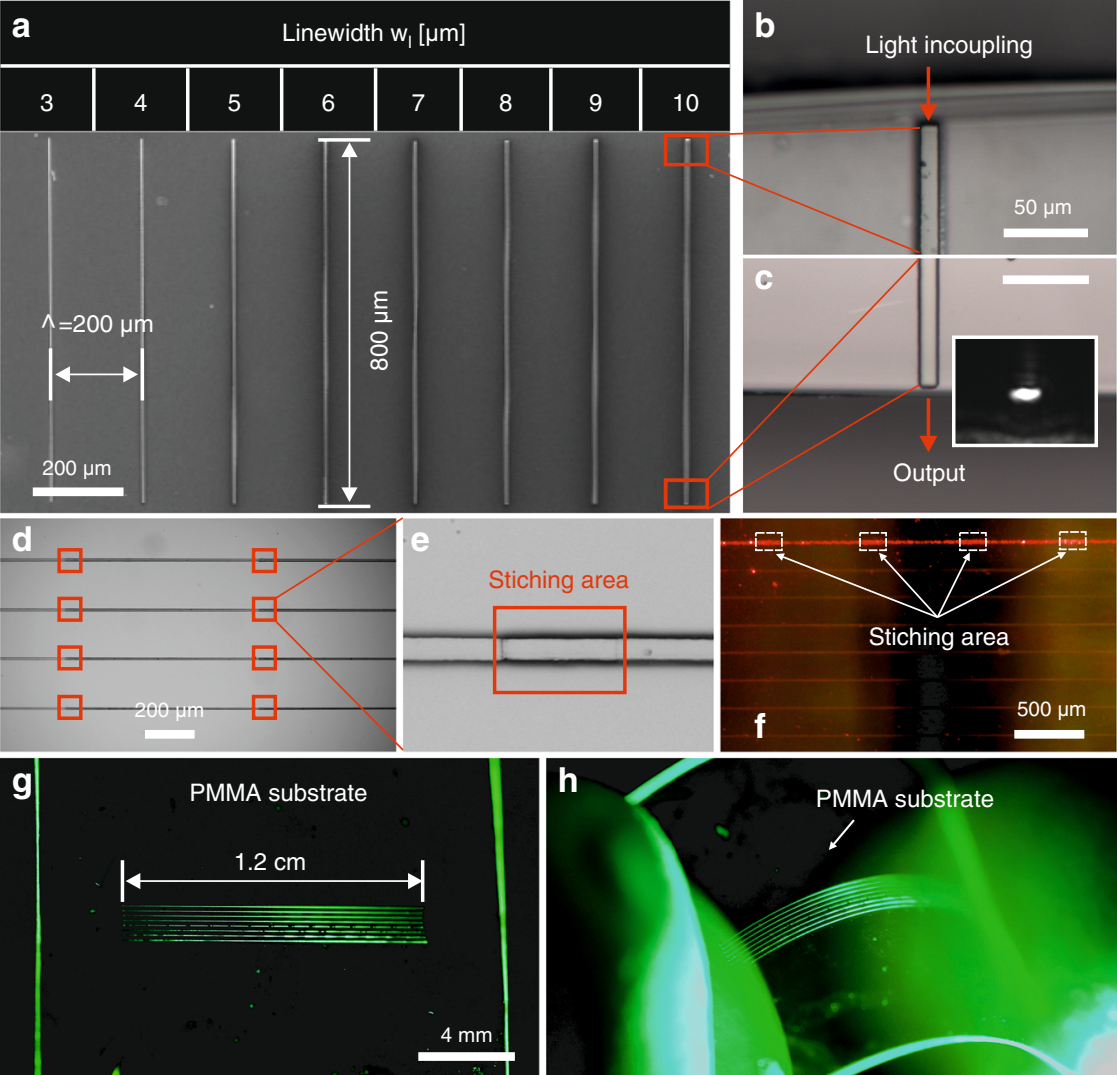
Multimode waveguides fabricated by MPP. **a** SEM image of multimode waveguides with the widths ranging from 3 µm to 10 μm (from left to right). **b, c** Microscope images of areas close to the ends of the waveguides (*w*_l_ = 10 μm), where the sample was cleaved. The output signal from one end of the waveguide is imaged by a CCD camera. **d** Microscope images of waveguides fabricated on a glass substrate by stitching eight parallel waveguide sections shown in (**a**). **e** Enlarged view of the stitching area where the photosensitive material was exposed twice. The width of the waveguide is 8 µm. The length of the stitching area is approximately 25 µm. **f** Bright-field microscope image of light guided through the waveguide (width: 10 µm). The doubly exposed area does not affect the propagation of light in the waveguide. **g** Long waveguides on a flexible PMMA substrate obtained by stitching eight parallel waveguide sections shown in (**a**). The length of each waveguide is approximately 1.2 cm. **h** Image of the long waveguides when bent.

In addition to fabrication of single-mode structures, a series of multimode waveguides with widths ranging from 3 µm to 10 μm were also realized (Fig. 4a). Each waveguide has a length of 800 μm and the space between two adjacent waveguides is Λ = 200 μm. They were made of a self-synthesized organic–inorganic hybrid photosensitive polymer^34^ deposited on a glass substrate. A 10x objective with a NA of 0.45 was used for fabrication. To verify the light-guiding performance, a waveguide with a width of 10 μm was used as an example. To efficiently couple light into the waveguide, the sample was cleaved at both sides very close to the ends of the structure. Microscope images of the cleaved areas are shown in Fig. 4b, c. A He–Ne laser beam producing 633 nm light was coupled into the waveguide, and the transmitted light was observed. The output light from the other end of the waveguide was imaged by a CCD camera (the bright spot of the dark-field image in Fig. 4c).

Furthermore, stitching of multimode waveguides with the structures shown in Fig. 4a was also performed on a glass substrate (see the microscope image in Fig. 4d). The red squares marked in the figure indicate the area where the photopolymer was exposed twice when two waveguides were stitched together. An enlarged observation image is shown in Fig. 4e, from which the stitched area can be clearly seen. The width of the waveguide is 8 µm, and the length of the stitched area is approximately 25 µm. The size of the stitched area can be controlled by setting the spatial parameters (e.g., the distance between two stitching structure arrays) before starting the fabrication process. Additionally, motorized stages with more precise movement would be helpful for improving the stitching accuracy. To demonstrate the functionality of the stitching waveguide, a laser beam (*λ* = 633 nm) was also coupled into one of those waveguides (width of the selected waveguide: 10 µm). Fig. 4e is a bright field microscope image showing the light guided through the waveguide with a few stitched areas. It seems that the doubly exposed area does not affect light propagation in the waveguide.

Additionally, structures composed of eight parallel long waveguides (Fig. 4g) were fabricated on a PMMA substrate through 15 stitches of the aforementioned multimode waveguides. Each long waveguide has a length of approximately 1.2 cm, and the spatial period of the structures is 200 μm. The widths of the waveguide range from 3 µm to 10 μm (from the bottom to the top). The substrate can be flexibly bent (Fig. 4h) without loss of performance.

### Microring resonators and waveguides containing laser active material

**Fig. 5:**
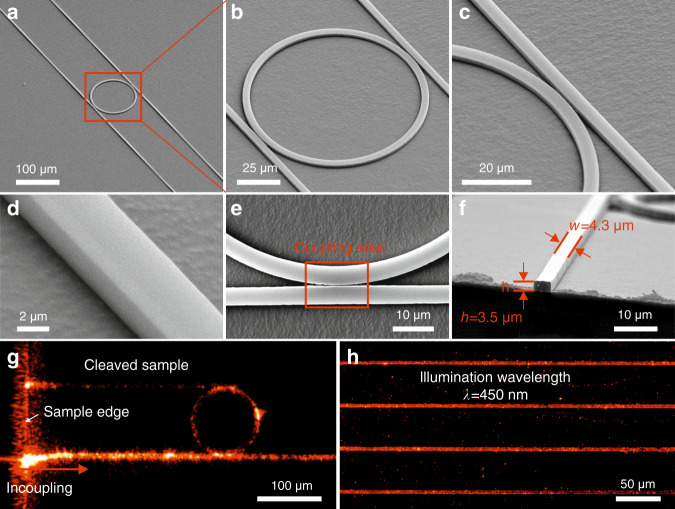
Microring resonators and waveguides, as well as demonstration of their functionality. **a–c** SEM images of a microring resonator with a diameter of 100 μm on a PMMA substrate. **d** Magnified view of the coupling waveguide, from which the surface smoothness can be seen. **e** Magnified SEM image of the microring resonator. A gap distance of approximately 370 nm was achieved between the resonator and waveguide. **f** SEM image of the cross-sectional view of a cleaved waveguide. The waveguide has a width of 4.3 μm and a height of 3.5 μm. **g** Microscope image of light propagation in a microring resonator when coupled by a laser beam at 633 nm. **h** Microscope image of polymeric waveguides made of OrmoCore embedded with CdSe/CdS core-shell type quantum rods and illuminated by a blue laser diode at 450 nm.

Two-dimensional microring resonators with two bus waveguides were fabricated on a PMMA substrate and SEM images are shown in Fig. 5a–c. The self-synthesized organic–inorganic hybrid photosensitive polymer^34^ and a 10x objective (NA = 0.45) were used for fabrication. The obtained structure exhibits good smoothness, as seen from the magnified SEM image of one bus waveguide (Fig. 5d). The microring resonator has a diameter of 100 μm in this case. The waveguide and the ring resonator are very close, with a gap distance of approximately 370 nm (Fig. 5e). A cross-sectional view of the cleaved waveguide used for coupling the light is shown in Fig. 5f. A clear and sharp section of the waveguide was realized. The measured width and height of the waveguide arm were 4.3 µm and 3.5 μm, respectively. For functionality demonstration, radiation from a He–Ne laser at 633 nm was coupled into the bus waveguide, with Fig. 5g illustrating the propagation of light in the structure. The optical signal leaks into the microring resonator and circulates inside it. Light coupled out from the ring resonator propagates in the opposite bus waveguide. The light scattering observed might result from tiny particles on the sample surface or residual surface roughness.

Finally, multimode waveguides were created using the polymer OrmoCore (micro resist technology GmbH) embedded with CdSe/CdS core-shell quantum rods, which exhibit amplified spontaneous emission (ASE) at a wavelength of approximately 623 nm^35^. The obtained waveguides were illuminated by a blue laser diode at approximately 450 nm. The dark-field microscope image (Fig. 5h) of the illuminated waveguides exhibits redshifted fluorescence consistent with the reported ASE of the doped material^35^. This result highlights the feasibility of realizing novel active optical components with doped nanomaterials.

## Discussion

The reported results, including micro- and nanostructuring with spatial periods of 400 nm and feature sizes down to 150 nm, large-area structuring with area sizes at the square millimeter and centimeter scales, fabrication on both rigid and flexible substrates, and especially, the structuring of polymers with embedded laser active materials, demonstrate the capabilities of UV-LED MPP in high-resolution and precise manufacturing. The developed system employs an inexpensive UV-LED instead of a mercury lamp as the light source and off-the-shelf commercially available optical components, so construction is simple and cost-effective. The development of the process chain (see description in the Materials and methods section) and autofocusing make fabrication user-friendly and efficient. Usually, single exposed structures can be produced within a few seconds. The implementation of a stitching process allows for large-area fabrication by overlapping or repeating reference structures (e.g., lines or gratings). With this fabrication technique, any type of structures, which can be printed on a transparent polymer foil and demagnified by a factor of up to three orders of magnitude depending on the applied microscope objective, can be fabricated. This means that a resolution on the micrometer scale on the printed transparent foil allows generation of structures with nanoscale resolution on the substrate. Thus, UV-LED MPP is suitable as a low-cost, highly efficient, and versatile tool for realizing functional optical micro- and nanostructures for various applications (e.g., sensing, data communication, and optical materials science) in the future. Additionally, the technique can be combined with etching processes to transfer the created patterns. Metallic structures can thus be fabricated, which is envisioned in subsequent steps.

## Materials and methods

### Materials

The materials used for the fabrication of structures include OrmoCore (micro resist technology GmbH) and a self-synthesized organic–inorganic hybrid photosensitive polymer exhibiting low shrinkage^34^. Commercially available OrmoCore is a hybrid polymer with preferred application in UV lithography. It offers an absorption coefficient of 113 cm^−1^ (approximately 29% absorbance) at a wavelength of 365 nm and shows glass-like optical properties (absorption coefficient is approximately 5 cm^−1^) within the wavelength range 550–1600 nm. The self-synthesized hybrid photopolymer exhibits a measured absorbance of approximately 32% at 365 nm in its liquid state^33^ and high transmission (approximately 99%) in the visible range^34,36^. Other photopolymers with sufficient absorption at the laser wavelength employed for structuring can also be adopted.

### Fabrication systems

Within this fabrication paradigm, we developed a process chain that covers the procedures from structure design to the final production process. Three main steps are included in this process chain: First, the desired structures are designed using standard vector-graphics software and printed on commercial transparent foil. The second step involves transferring the printed structures lithographically with a demagnification of 10:1 onto a chromium photomask using a developed TPP setup. The third step involves using the structure-patterned chromium photomask in the developed UV-LED MPP setup to demagnify and project structure images with a commercially available microscope objective onto a photosensitive substrate. The structure patterns on the transparent foil can be demagnified with a ratio of 1000:1 when a 100x microscope objective is used.

#### Structure design and pattern printing

The desired micro- and nanostructures were first designed with vector-graphics software, in this case CorelDraw. The graphs were then printed onto photosensitive silver layers embedded in a transparent polymer foil using a laser plotter. This plotter has a resolution of 1.65 µm (16,256 dpi), offering an effective resolution of 10 µm to the printed structures. The theoretical minimum feature size that can be achieved is then determined by the imaging quality of the lithography systems and the microscope objective employed.

#### Tessar projection photolithography of a patterned chromium photomask

**Fig. 6:**
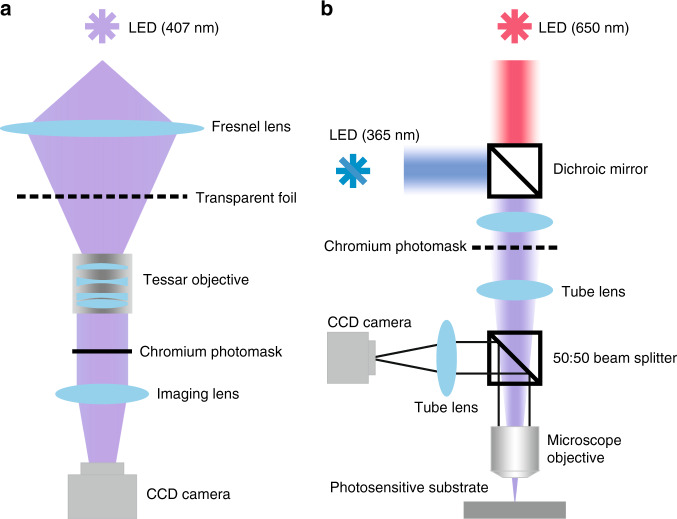
Schematic illustrations of the developed experimental setups. **a** Tessar projection photolithography. **b** Microscope projection photolithography setup.

Tessar projection photolithography was developed to transfer the printed structure patterns onto chromium photomasks after the printing process. The TPP setup, as illustrated in Fig. 6a, is simple and only consists of several components. A high-power UV-LED (*λ* = 407 nm, Φ_*e*_ = 4.9 W optical output power), which was placed at the focal plane of the Fresnel lens, was employed for the exposure of the photopolymer coated on the chromium mask below. A Fresnel lens was used to collect the light radiation emitted by the UV-LED. The printed transparent polymer foil was placed in the back focal plane of a 10x Tessar objective with a 50 mm focal length, which demagnified the structure patterns on the foil by ten times onto the intermediate chromium photomask placed at its focal plane. A fresh chromium mask was prepared by covering a 100 nm thick chromium layer onto a BK-7 glass substrate with a diameter of 25.4 mm and thickness of 1 mm and subsequently spin-coating a wet-etch-resistant photopolymer (S1805, micro resist technology GmbH) layer onto the chromium layer. A CCD camera (Imaging Source 72bXYZ) was installed below to image the focal plane of the Tessar objective to assist the focusing adjustment of the objective and the chromium photomask surface. A development process followed the irradiation of the photopolymer. Subsequently, the chromium photomask was placed into the chromium etchant (Chrome Etch N°1, MicroChemicals GmbH) to etch the imaged structures into the chromium layer. Thus, a photomask with a structural pattern was obtained. In addition, acetone can be used to wash away the remaining photopolymer, and deionized water can be used to further clean the photomask.

#### Microscope projection photolithography for fabrication of micro- and nanostructures

In the final step, MPP was employed for the generation of micro- and nanostructures. The setup (Fig. 6b) was developed mainly with commercially available optical components.

A high-power UV-LED (*λ* = 365 nm, Φ_*e*_ = 693 mW optical output power) collimated using an aspheric lens was mainly employed for the exposure process. This is much cheaper than a standard mercury lamp employed for projection photolithography. A red LED (*λ* = 650 nm, Φ_*e*_ = 40 mW optical output power), which does not cure UV-sensitive photopolymers, was used for the focusing process, by which the photosensitive substrate surface and the projected image of the photomask were synchronized at the focal plane of the microscope objective. The light of the red LED was also collimated by an aspheric lens and propagated along the same beam path as that of the UV-LED. A prepared photomask with the desired structure pattern was placed in the image plane of the optical microscope objective. Through light illumination, the structure image on the photomask can be projected to a tube lens below (Fig. 6b). Within the area of the photomask (25.4 mm) in this setup, the drop in illumination light intensity toward the edge is below 0.5%, which can be neglected. Therefore, the illumination intensity is assumed to be uniform. As a consequence, the intensity distribution on the projected structure pattern is homogeneous. The tube lens was mounted at a distance of 16.45 cm to the patterned photomask to project the structure image into infinite space, which was created by the combination of the tube lens and the microscope objective. This configuration minimizes chromatic aberrations in the image plane and ultimately produces a flat structure image across the field of view on the substrate. Here, plan-apochromat microscope objectives with color correction and field flatness were used. Microscope objectives with different magnifications can be used to reach the desired demagnified scaling of the image on the substrate surface, which was then reflected to a beam splitter that was integrated with a tube lens within the infinity space to project the reflected structure image on the substrate onto a computer-connected CCD camera. With this configuration, the projected demagnified image on the substrate surface was visualized on a computer screen.

By varying the z-axis position of the stages, structure images with different sharpness were observed. The position where the visualized image shows the best edge sharpness is considered the focus position. This focusing process can usually be manually operated. However, it is relatively time-consuming and not user-friendly enough. Therefore, we developed an autofocusing program, which is realized by computational image processing based on edge detection of structure images^37^ and enables us to find the focus position in a few seconds. During the autofocusing process, the z-position of the stage was automatically varied using a computer-controlled motor. Furthermore, the structure images projected from the patterned photomask onto the substrate at different z-positions were recorded by the CCD camera, and the information of each structure image was compared using the computer program. The pixel information of images was processed with the help of the Canny algorithm^37^, which works based on edge detection. The position where the image exhibits the best contrast of edge area to the neighboring area and maximum edge strength is considered to be the correct focus position. In the case of focusing a photosensitive substrate, only the red LED was on during the autofocusing process to project the structure image on the photomask to the substrate surface, while the UV-LED was switched off to avoid precuring the photopolymer on the substrate.

Although chromatic aberration was minimized by employing a tube lens and apochromatic microscope objectives, minor offset of focus positions between the UV-LED and red LED still existed. This offset was usually within a range of a few microns and was easily measured by subtracting the absolute focus position of the z-stage, where the projected structure image was focused on a substrate, under red LED illumination from that under UV-LED illumination. The absolute focus positions were obtained using the autofocusing process. With compensation of the measured offset value in our computer program, the focus position was adjusted accordingly without the need to perform additional autofocusing under UV illumination when the light source was changed from red to UV-LED for structuring of the photosensitive material on the substrate.

In addition to automatically focusing the substrate surface onto the focal plane of the microscope objective, a triangulation process for the alignment of the substrate along both the X and Y directions can also be performed based on the autofocusing program when necessary. During the triangulation, the stage moves to three selected points, which form a right triangle within the area of the applied substrate with two points on the x-axis and two points on the y-axis, and the focus position at each selected point is determined based on the autofocusing program. If the three obtained focus positions are different, the substrate is considered to be tilted. Thus, it is necessary to adjust the tilt of the stage by rotating the knobs for tilt control on each axis. This procedure should be performed until the same focus positions are obtained at those three points, and the substrate is considered well aligned.

A motorized XY scanning stage with a travel range of 102 mm × 102 mm and a step resolution of 2.5 µm was used to move the photosensitive sample. The fabrication area is limited by the field of view of the applied microscope objective in the case of single-step exposure. The minimum achievable feature size is determined by the NA of the objective. Table 2 shows the estimated field of view, lateral resolution, and depth of field of the microscope objectives employed in this work. The lateral resolution and depth of field were calculated using formulae $\frac{\lambda }{{2NA}}$ and $\frac{{n\lambda }}{{NA^2}}$, respectively, where *λ* indicates the wavelength and *n* is the refractive index between the objective and the coverslip^38,39^. According to the results, a 10x objective with a NA of 0.45 can be used to expose a circular area with a diameter of 2.5 mm and achieve a theoretical minimum feature size of 405 nm. On the other hand, a 100x objective with a NA of 1.4 enables the reduction of the exposed area to a diameter of 250 µm, and the minimum feature size that can be realized is also reduced to 130 nm.

**Table 2:** Lateral resolution and depth of field of the objectives employed in this work.

Magnification	NA	Field of view [mm]	Lateral resolution [nm]	Depth of field [µm]
10x	0.45	2.5	405	1.8
100x	0.95	0.25	190	0.4
100x	1.4	0.25	130	0.28

In this work, a stitching process was developed to perform large-area structuring (see large-area structures demonstrated in the Results section), which is realized by overlapping or repeating reference structures. The spatial parameters, such as the gap distance between adjacent reference structures and the numbers of reference structures in the X and Y directions, can be set to control the fabrication process and achieve the desired large-area structures.

## Data Availability

The data supporting plots within this paper and other findings of this study are available from the corresponding author upon request.
